# AUTO-SP: Automated Sample Preparation for Analyzing Proteins and Protein Modifications

**DOI:** 10.1021/acs.analchem.5c00886

**Published:** 2025-07-28

**Authors:** T. Mamie Lih, Liyuan Jiao, Lijun Chen, Jongmin Woo, Yuefan Wang, Hui Zhang

**Affiliations:** † Department of Pathology, 1500 Johns Hopkins University School of Medicine, Baltimore, Maryland 21231, United States; ‡ Department of Oncology, Sidney Kimmel Cancer Center at Johns Hopkins Medical Institutions, Baltimore, Maryland 21231, United States; § Department of Urology, Johns Hopkins University School of Medicine, Baltimore, Maryland 21231, United States

## Abstract

Liquid chromatography (LC) tandem mass spectrometry (MS/MS) is one of the widely used proteomic techniques to study the alterations occurring at the protein level as well as post-translational modifications (PTMs) of proteins that are relevant to different physiological or pathological statuses. The mass spectrometric analysis of peptides digested from proteins (bottom-up proteomics) has emerged as one of the major approaches for proteomics. In this approach, proteins are first cleaved by one or more proteases into peptides for MS analysis, and peptides with PTMs are further enriched, followed by the LC-MS/MS analysis. To achieve a reproducible and quantitative proteomic characterization, a well-established protease digestion and PTM peptide enrichment protocol is critical. In this study, we developed AUTO-SP, a sample preparation platform providing automated protocols for BCA analysis, protein digestion, and PTM enrichment for protein and PTM analyses. We utilized patient-derived xenograft (PDX) breast cancer tumor tissues (basal-like and luminal subtypes) to demonstrate the efficacy of AUTO-SP. The protein amount was quantified, and proteins were further digested by using AUTO-SP for each PDX sample. Based on the data-independent acquisition (DIA)-MS data, we observed that samples of the same breast cancer subtypes were highly correlated (≥0.98). Additionally, >25,000 phosphopeptides and >14,000 ubiquitinated peptides were identified in the PDX samples when using AUTO-SP for PTM enrichment, while unique pathways were enriched from the differentially expressed ubiquitinated peptides of basal-like and luminal subtypes. AUTO-SP demonstrated its efficacy to provide a reliable and reproducible sample preparation procedure for MS-based proteomic and PTM analyses.

## Graphical abstract

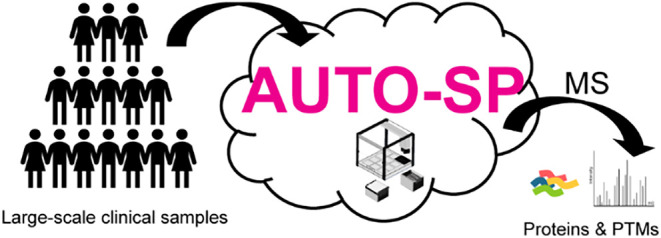

## Introduction

Quantitative proteomic analysis via mass spectrometry (MS) is one of the widely adopted techniques to study the alterations occurring at the protein level as well as aberrant post-translational modifications (PTMs) of proteins that are relevant to the development of diseases.^1–5^ Moreover, recent advancements of MS have enabled large-scale proteomic studies of several cancer types, expanding our understanding of the molecular basis of these cancers.^1–3,6–26^ To successfully conduct MS-based quantitative proteomic and PTM studies via LC-MS/MS, the samples are prepared by protease digestion to obtain global peptides, while PTM-containing peptides require further enrichment.^27–29^ Sample preparation can impact nearly all of the later steps in a proteomic study. Therefore, it is critical to design and establish a sample preparation protocol that is robust and reproducible to ensure the scientific integrity of the study. Additionally, a sample preparation workflow with high-throughput capability would be ideal when processing large sets of samples.

The Clinical Proteomic Tumor Analysis Consortium (CPTAC) established and reported a sample preparation protocol for deep-scale MS-based proteomic and phosphoproteomic analysis in 2018 ^27^ (referred to as 2018 CPTAC protocol). This protocol has been used in various CPTAC-related studies, while the data generated from those studies have been publicly available for the broad scientific community.^1,2,6–9^ In this study, we developed AUTO-SP, an automated sample preparation platform for analyzing proteins and protein modifications. We transformed the essential steps in the 2018 CPTAC protocol into automated procedures using an automated liquid handling system, including protein concentration measurement via the bicinchoninic acid (BCA) assay, protein digestion, and phosphopeptide enrichment via immobilized metal affinity chromatography (IMAC) magnetic beads. In addition to IMAC enrichment, our enrichment protocol allows enriching other PTMs, such as ubiquitination, acetylation, and phosphotyrosine, which were not included in the 2018 CPTAC protocol. By applying automation, we would be able to increase sample throughput and reduce any potential human errors to enhance the reproducibility and consistency in sample preparation.

In this study, we utilized patient-derived xenograft (PDX) breast cancer tumor tissues from mouse models of two subtypes, P96 (basal-like) and P97 (luminal), to evaluate the AUTO-SP. Using AUTO-SP, we ran BCA analysis on 8 PDX pooled samples and achieved consistent results, where the coefficient of variation (CV) for each sample was below 5.5%. The proteins from each sample were further enzymatic digested using AUTO-SP in a 96-well plate. Based on the data-independent acquisition (DIA)-MS data, we observed that samples of the same breast cancer subtypes were highly correlated (≥0.98) with missed cleavage rate between 6 and 7.5%. Furthermore, >25,000 phosphopeptides and >14,000 ubiquitinated peptides were identified in the PDX samples when using the PTM enrichment protocol of the AUTO-SP. Additionally, we found that unique pathways were enriched from differentially expressed ubiquitinated peptides of the P96 and P97. Taken together, AUTO-SP demonstrated its efficacy to provide a reliable and reproducible sample preparation procedure for MS-based proteomic and PTM analyses.

## Experimental Section

### Tissue Samples

The PDX breast cancer tumor tissues from mouse models, P96 (basal-like) and P97 (luminal), were used in this study. All tumor pieces were cryopulverized and stored at −80 °C until sample preparation for the analysis of global proteomic and protein modifications. The details for preparing bulk cryopulverized PDX tissues can be found in our previous publication.^27^

### Sample Processing for Protein Extraction, Protein Digestion, and PTM Enrichment

In this study, BCA analysis, protease digestion, and enrichment of ubiquitinated and phosphopeptides were carried out by using AUTO-SP, and details can be found in the Results. Tissue lysis was performed as previously described.^27^ In brief, 400 μL of urea lysis buffer (8 M urea, 75 mM NaCl, 50 mM Tris, pH 8.0, 1 mM EDTA, 2 g/mL aprotinin, 10 g/mL leupeptin, 1 mM PMSF, 10 mM NaF, Phosphatase Inhibitor Cocktail 2 and Phosphatase Inhibitor Cocktail 3 [1:100 dilution], and 20 mM PUGNAc) was added to 100 mg of each cryopulverized PDX tissue, followed by repeated vortexing. Lysates were clarified by centrifugation at 20,000*g* for 10 min at 4 °C. Lysates from the same breast cancer subtype were pooled together before measuring protein concentrations by the BCA assay (Pierce). For the protease digestion, pooled samples were aliquoted into a 96-well plate, and each well contained 1 mg of protein. In each well, proteins were reduced with 5 mM dithiothreitol (DTT), alkylated with 10 mM iodoacetamide (IAA), diluted 1:3 with 50 mM Tris-HCI (pH 8.0), digested with Lys-C (Wako Chemicals) at a 1 mAU:50 g enzyme-to-substrate ratio, and sequencing-grade modified trypsin (Promega) at a 1:50 enzyme-to-substrate ratio. The digested samples were then acidified with 50% formic acid (FA, Sigma) to a pH value of approximately 2.0. Tryptic peptides were desalted on a 100 mg Sep-Pak C18 SPE plate (Waters). To examine the digestion efficiency, 1 μg of digested peptides was aliquoted from 12 randomly selected wellsε. All digested samples were dried in a Speed-Vac. Magnetic Fe-NTA beads (Cube Biotech) were used to enrich the phosphopeptides. Ubiquitinated peptides were enriched using antibody-based magnetic beads from the PTMScan HS Ubiquitin/SUMO remnant motif (K-ε-GG) kit (Cell Signaling Technology).

### LC-MS/MS Analysis

All of the LC-MS/MS data were acquired via an Evosep One EV-1000 (EVOSEP) coupled with a timsTOF HT (Bruker) in DIA mode. The methods for acquiring global peptides, phosphopeptides, and ubiquitinated peptides are as follows. A PepSep C18 column of 15 cm × 150 μm (1.5 μm, Bruker) was used for peptide separation of 30 samples per day (30 SPD: 44 min gradient length, 0.5 uL/min flow rate, LC gradient from 3 to 35%) at 50 °C. The data were acquired under the dia-PASEF mode with a MS1 scan range of 100–1700 *m*/*z*, MS2 scan range of 338.6–1338.6 *m*/*z*, 1/K0 range of 0.70 to 1.45 V/s/cm^2^, and Ramp time of 85 ms. The MS2 scan range was 395.7–1645.7 *m*/*z* for phosphopeptides and 341.6–1216.6 *m*/*z* for ubiquitinated peptides, while other parameters were the same as those of the global peptides.

### MS Data Analysis

All of the raw files were searched against a UniProt/Swiss-Prot database containing human and mouse proteins (downloaded on 2019/12, 37,405 entries) using the directDIA approach in the Spectronaut (version 18.5, Biognosys). The search setting is as follows. Mass tolerance of MS and MS/MS was set as dynamic with a correction factor of 1. Precursors were filtered by a *Q* value cutoff of 0.01 (which corresponds to a FDR of 1%). Carbamidomethyl (C) was set as a fixed modification. Acetyl (Protein N-term) and Oxidation (M) were set as variable modifications. Variable modifications of Phospho (STY) and GlyGly (K) were set additionally for searching phosphopeptides and ubiquitinated peptides, respectively. For global protoemic data, the quantity of a protein was the sum of the quantity of its top 3 peptides (stripped sequences). For PTM data, the quantity for a peptide (modified sequence) was calculated by summing the quantity of its top 3 precursors.

Reproducibility among the triplicates was determined based on the Spearman correlation. Specificity of the enrichment of PTMs was calculated by summing the abundances of all modified peptides and then dividing by the total abundances of all identified peptides. Differential analysis was carried out by calculating the median log 2 fold changes between two breast cancer subtype groups, and a two-sided *t* test was performed with the *p*-value adjusted via the Benjamini–Hochberg method. The KEGG pathways were enriched using the over-representation analysis on the WebGestalt (version 2019).^30^

## Results

### Overview of AUTO-SP

To increase throughput, minimize human errors, and reduce time and cost for large-scale sample preparation, we have established AUTO-SP, which currently can automate BCA assay analysis, in-solution protein digestion, and magnetic bead-based enrichment for various protein modifications, including ubiquitination and phosphorylation. Figure 1 illustrates sample preparation protocols available on AUTO-SP. Tumor tissues from the PDX models (P96 and P97) were used to demonstrate the feasibility and reproducibility of the established automated sample preparation platform for the analyses of proteins and protein modifications. For demonstration purposes, we only showed the results of automated enrichment of phosphopeptides and ubiquitinated peptides in this study. Of note, we used the Opentrons OT-2 to demonstrate the AUTO-SP in this study; however, we have established Opentrons Flex-compatible protocols as well. The protocols were written using Python and executed via the Opentrons software (version 6.3.1) to control the instrument. The protocols are available in the Supporting Information file.

**Figure 1:**
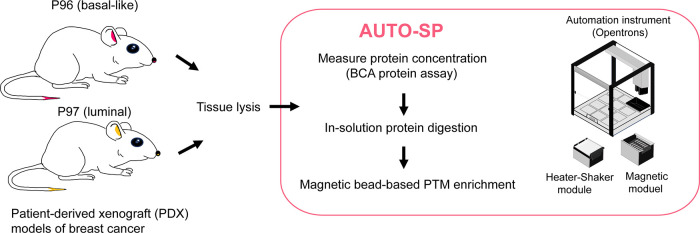
Sample preparation procedures using AUTO-SP. The amount of proteins in PDX tumor tissues was measured using the BCA protocol on AUTO-SP, followed by protease digestion. Phosphopeptides and ubiquitinated peptides were enriched from the global peptides obtained using the AUTO-SP.

### Evaluation of Automated BCA Analysis for Protein Concentration Measurement

Prior to protein digestion, measuring protein concentration in a sample is essential to ensure adding an adequate amount of protease (e.g., trypsin) to avoid incomplete or over-digestion of proteins. Using the BCA protein assay to quantify protein amount is a widely adopted approach in proteomic studies.^28^ BCA reagent is added and incubated with diluted samples before the protein concentration is read in each sample. A triplicate of each sample is usually used to ensure the accuracy of measurement. The whole process is simple and easy to operate when handling only a small set of samples. However, large-scale proteomic studies could have more than 100 samples that need to be analyzed. Manually processing BCA assays will then become time-consuming, and human errors are likely to occur when transferring samples from individual vials to a 96-well BCA assay plate. Therefore, we established the automated BCA assay protocol to increase throughput while reducing human and random errors.

After urea-based tissue lysis, pooled P96 and pooled P97 were diluted using HPLC water (1:20 ratio) and placed into a 96-well plate (i.e., Source plate) along with the BSA protein standards (Figure 2A). Figure 2B demonstrates the layout on OT-2 for this protocol. The BCA protocol can be started when all required labware is placed in the designated slots. As shown in Figure 2C, the measured protein concentration was consistent between pooled P96 samples and among pooled P97 samples. Moreover, the majority of the samples with CV less than 3% among the triplicates were observed, while the maximum CV was 5.08% (Figure 2D), further indicating the successful execution of the automated BCA analysis.

**Figure 2:**
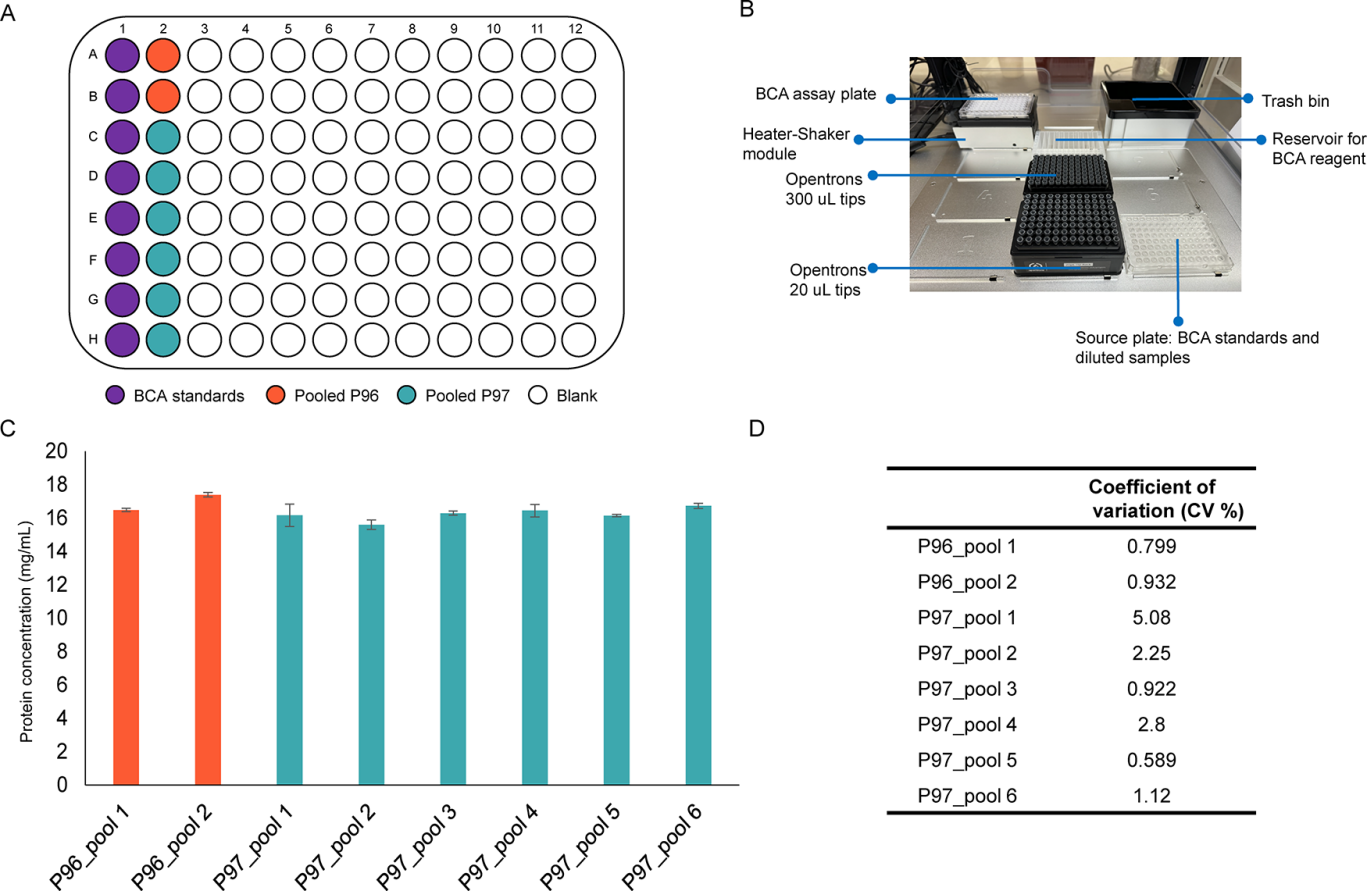
BCA analysis of pooled samples. (A) Layout on a 96-well plate (Source plate) was used for holding BCA standards and diluted samples for the current study. Each well of the BCA standard contained 30 μL, while the wells for the diluted samples contained 40 μL for each. Each BCA standard was transferred twice (two replicates), and each diluted sample was transferred three times (triplicate) from the Source plate to the BCA assay plate on the Heater-Shaker module. (B) Layout of the labware on the automation platform for BCA analysis. (C) Protein concentration was measured in each pool sample. (D) CVs were calculated in triplicate for each pool sample.

### Assessment of In-Solution Digestion

For the bottom-up proteomics, proteins and protein modifications are identified and quantified based on the peptides derived from each protein by enzymatic digestion. The amount of a protease to be added is estimated according to the results obtained from the BCA analysis, as described above. In this study, we used Lys-C and trypsin for protein digestion; however, the protocol can be adapted to protein digestion using other proteases.

The pooled P96 and P97 were distributed to a 96 deep-well plate (Source plate) in which each well contained approximately 1 mg of proteins (Figure S1A). The layout of the labware on the automation platform was similar to the BCA analysis, but the Source plate was placed on the Heater-Shaker module instead. All of the reagents, buffer, and proteases were added automatically to the source plate when executing the digestion protocol via AUTO-SP. The incubation of IAA (45 min), Lys-C (2 h), and trypsin (overnight, ∼16 h) was carried out on the Heater-Shaker at room temperature (∼25 °C) at a speed of 500 rpm, except for the DTT, which required incubation at 37 °C. Transferring and incubation of IAA were performed in the dark. Note that the Heater-Shaker module provides heat at the surface of its top plate. Therefore, protocols requiring precise temperature control demand extra attention.

We randomly selected 12 wells to evaluate the efficiency of AUTO-SP on in-solution digestion (Figure S1A and Table S1). High reproducibility was observed among the wells containing the same sample types since the Spearman correlation was ≥0.98 (Figures 3A and S1B) with median CV below 10% for P96 and P97 (Figures 3B and S1C), indicating that AUTO-SP could provide consistent results. Moreover, the missed cleavage rate was between 6.1 and 7.5%, regardless of the sample types (Figure 3C). The total number of peptides and proteins identified from P96 and P97 samples is as shown in Figure 3D. On average, 106,700 peptides and 11,700 protein groups were identified across P96 and P97. Additionally, we found a linear relationship between manual procedure and AUTO-SP for enzymatic digestion (Figure S1D) with the Spearman correlation ranging from 0.86 to 0.88 based on the global protein expression (Table S2). Overall, AUTO-SP produced consistent and reliable results while supporting high throughput of protein digestion.

**Figure 3:**
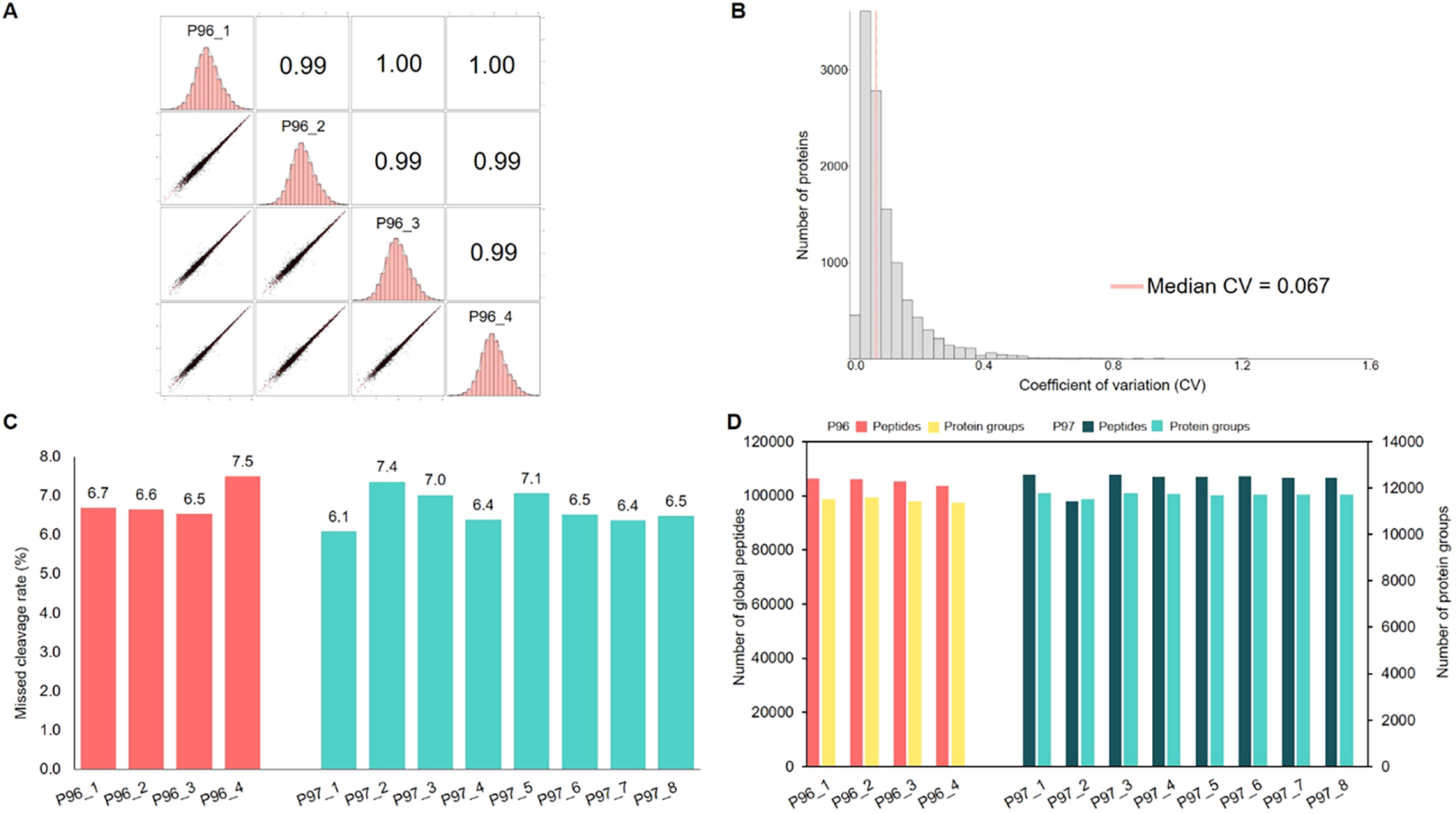
In-solution protein digestion was performed using AUTO-SP. (A) Reproducibility of protein digestion on AUTO-SP for P96. (B) CV of proteins identified in P96 samples. (C) Missed cleavage rate for each sample. (D) Total identified global peptides and protein groups for each sample.

### Evaluation on Phosphopeptide Enrichment via AUTO-SP

In addition to BCA analysis and in-solution protein digestion, AUTO-SP also facilitates enrichment of protein modifications via magnetic beads, accommodating both antibody-based and nonantibody-based beads. Phosphorylation is one of the most prevalent PTMs of proteins, which is involved in many cellular processes, such as cell cycle progression, metabolism, and apoptosis.^31–33^ Phosphopeptide enrichment using IMAC with Fe^3+^-loaded NTA agarose beads was described in the 2018 CPTAC protocol.^27^ In this study, we used IMAC with magnetic Fe-NTA beads to facilitate an automated PTM enrichment process on AUTO-SP.

Figure 4A shows the layout used for the magnetic bead-based PTM enrichment on AUTO-SP. In brief, the global peptides generated using the AUTO-SP digestion protocol were pooled and used for phosphopeptide enrichment. Pooled global peptides (∼100 μg peptides) from each breast cancer subtype were aliquoted into a 96-well plate (8 wells for each subtype in the Source plate). A separate 96-well plate containing magnetic Fe-NTA beads (15 μL bead slurry in each well) was placed on the magnetic module (i.e., Mag plate). The PTM enrichment protocol on AUTO-SP included the following: (1) transferring samples from the Source plate to the Mag plate, (2) incubating samples with the beads (3 cycles), (3) taking flow-through, (4) washing beads with 80% ACN/0.1%TFA (3 cycles), and (5) eluting phosphopeptides from the beads (100 μL 500 mM potassium phosphate buffer pH 7 twice with final total volume of 200 μL). Pipette mixing was used to thoroughly mix the samples with the beads during incubation and elution, as well as during bead washing. Eluted phosphopeptides were collected into the Collection plate (Figure 4A).

**Figure 4:**
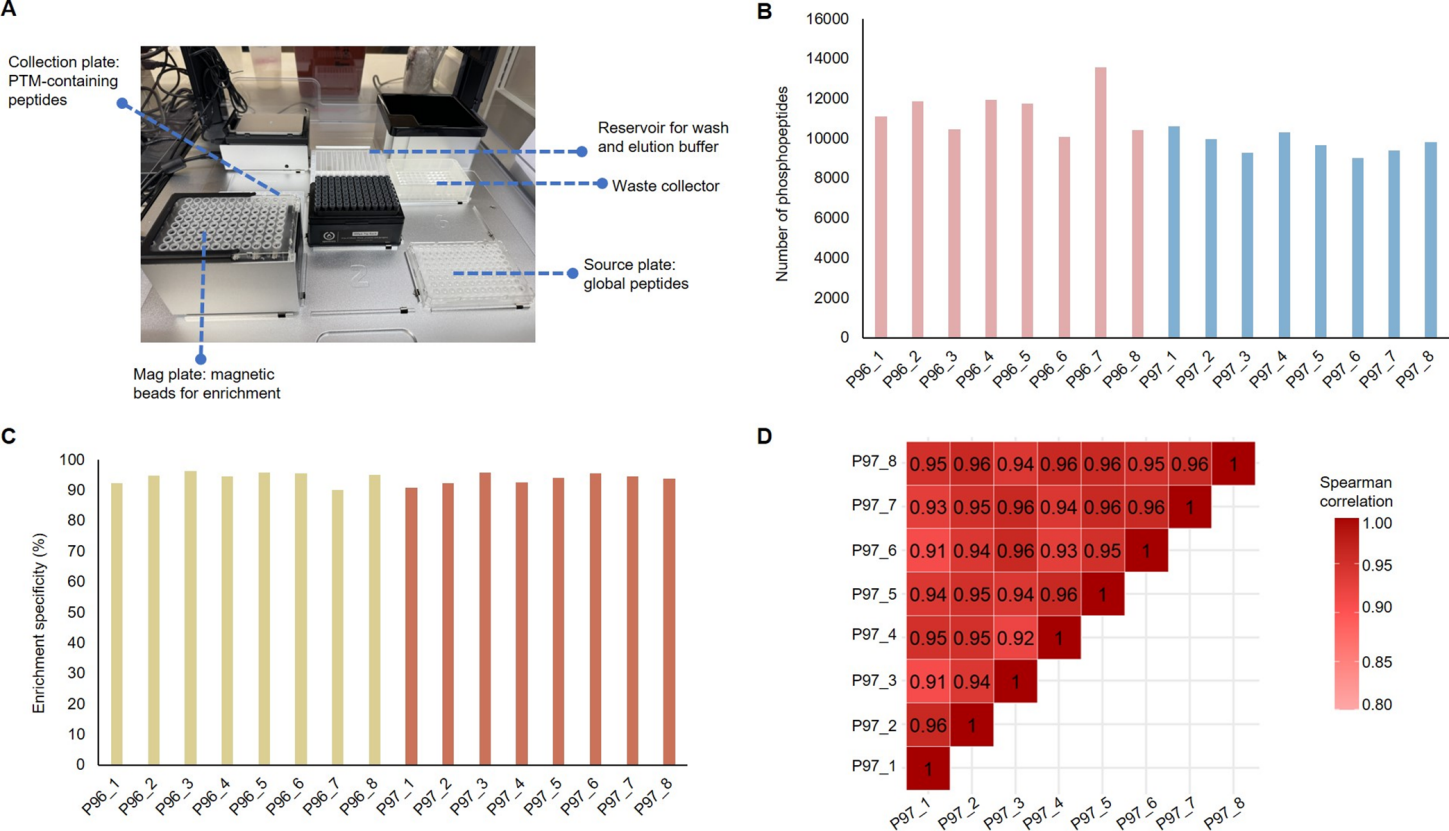
Phosphopeptide enrichment using AUTO-SP. (A) Layout of labware for magnetic bead-based phosphopeptide enrichment. (B) Number of identified phosphopeptides in each sample. (C) Phosphopeptide enrichment specificity of each sample. (D) Reproducibility of the phosphopeptide enrichment was determined across P97 samples.

Using the AUTO-SP, we were able to enrich >25,000 phosphopeptides across the samples (Table S3). The number of enriched phosphopeptides was different among the samples but similar within the same subtype (Figure 4B). Moreover, we found that the enrichment using AUTO-SP was effective since it could achieve the enrichment specificity ≥90% (Figure 4C). The reproducibility among the replicates was adequate, with the majority showing a Spearman correlation ≥0.9 for both P96 and P97 (Figures 4D and S2A). We also observed a good quantification stability based on the standard deviation of the abundances of phosphopeptides across samples of P96 or P97 (Figure S2B). The association between AUTO-SP and manual enrichment using magnetic beads was linear and strongly correlated (Figure S2C and Table S4). Taken together, AUTO-SP demonstrated its ability to enrich phosphopeptides with high enrichment specificity and provide reproducible results while performing similarly to the manual procedure.

### Magnetic Bead-Based Ubiquitin Antibody Enrichment Analysis

Enrichment of PTMs other than phosphopeptides was not included in the 2018 CPTAC protocol. We showed that AUTO-SP could enrich phosphopeptides efficiently using the nonantibody-based magnetic beads as described above. Here, we demonstrated the efficacy of protein modification enrichment on AUTO-SP using antibody-based magnetic beads to isolate ubiquitinated peptides from P96 and P97.

We first pooled the global peptides generated from the AUTO-SP and aliquoted them into 6 replicates for each breast cancer subtype. Each replicate started with approximately 300 μg of peptides and 5 μL of magnetic bead slurry. The enrichment process on AUTO-SP was similar to the phosphopeptide enrichment, except the beads were washed using IAP wash buffer (2 cycles), followed by PBS (2 cycles), and ubiquitinated peptides were eluted from the beads with 0.15% TFA (100 μL for 2 cycles). Pipette mixing was also used during incubation, washing, and elution steps.

By using the magnetic bead-enrichment protocol on the AUTO-SP, a total of 16,531 nonredundant ubiquitinated peptides were identified from 6 samples of P96, with an average identification of 14,770 ubiquitinated peptides (Figure 5A and Table S5). The enrichment specificity ranged from 19 to 34%, with a median of 24.8% (Figure 5B). Similarly, 16,701 ubiquitinated peptides were identified across the replicates of P97 (Table S5) with an enrichment specificity ranging from 23.7 to 33.7% (median = 28.7%). Additionally, we observed good quantification stability (Figure S3A,B).

**Figure 5:**
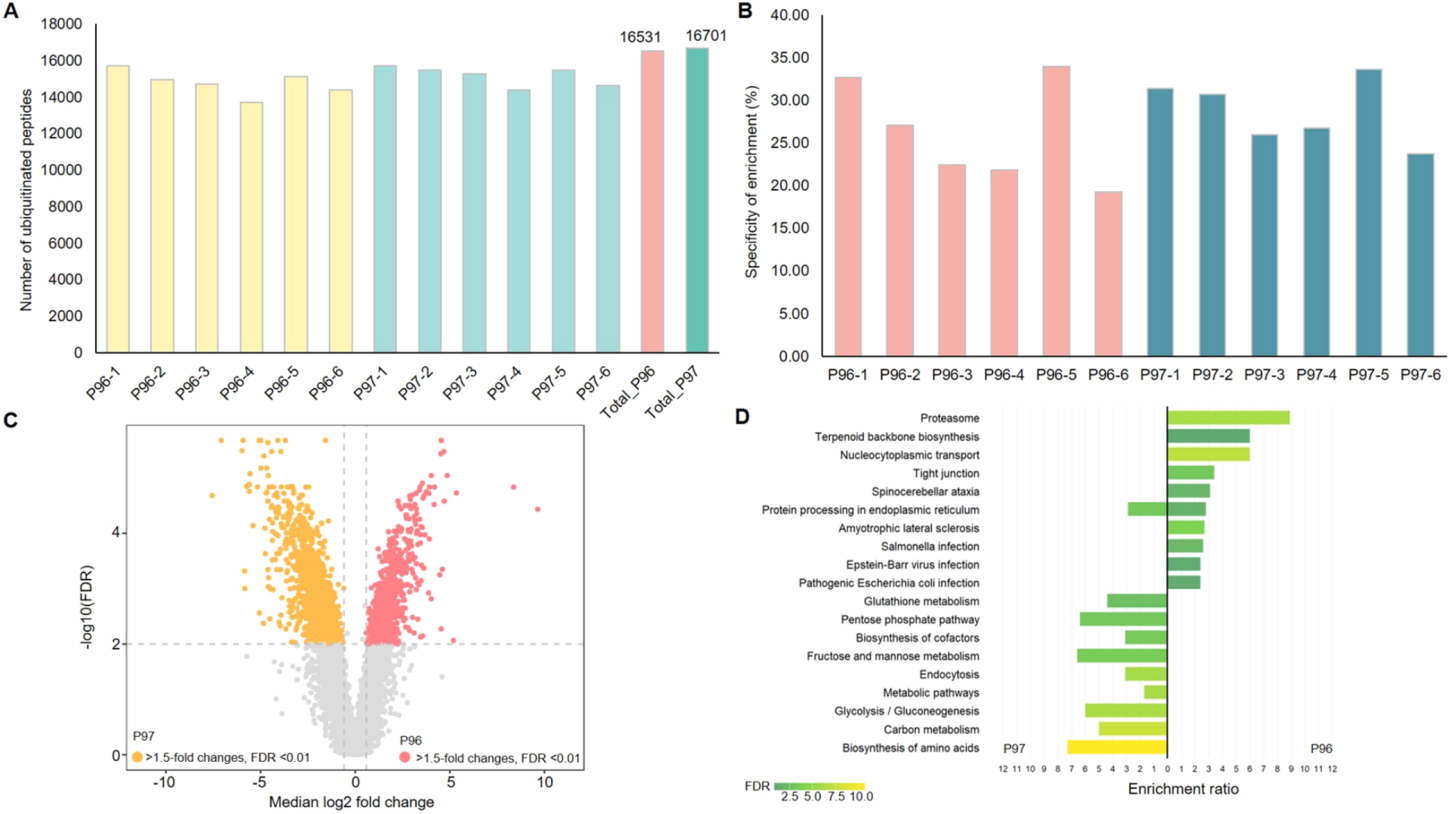
Ubiquitin enrichment via AUTO-SP. (A) Number of identified ubiquitinated peptides in each sample and total nonredundant ubiquitinated peptides identified across P96 and P97. (B) Ubiquitin enrichment specificity. (C) Comparison of P96 and P97 was based on the enriched ubiquitinated peptides (human only). (D) Enriched KEGG pathways were based on unique proteins with ubiquitinated peptides that were differentially expressed in P96 and P97.

Ubiquitination of proteins regulates various cellular processes, including proteasomal degradation, intracellular trafficking, and DNA repair.^34,35^ An association between cancer development and dysregulation of protein ubiquitination has been reported previously.^34,36,37^ Comparing the two PDX breast cancer subtypes, 719 ubiquitinated peptides (originating from 421 human proteins) with elevated expression in P96 relative to P97. On the other hand, 1,293 ubiquitinated peptides (originating from 573 human proteins) with higher abundance in P97 than P96 (Figure 5C and Table S6). From the differentially expressed ubiquitinated peptides, we found that unique pathways were enriched for P96 and P97 (Figure 5D). For example, the nucleocytoplasmic transport pathway emerged from P96, while the glycolysis/gluconeogenesis pathway was enriched in P97. In this study, two ubiquitinated peptides of nuclear pore complex protein Nup153 (NUP153) showed a more than 2-fold increase in P96 relative to P97. NUP153 is part of the nuclear pore complexes and is enriched in the nucleocytoplasmic transport pathway in this study. Others have found that NUP153 is abnormally expressed in prostate cancer, colorectal cancer, and thyroid cancer.^38–40^ Depletion of NUP153 could regulate cancer cell growth in a human breast cancer cell line.^41^ Phosphoglycerate kinase 1 (PGK1) was found to be enriched in the glycolysis pathway in the current study, and 6 of its ubiquitinated peptides were overexpressed in P97 compared to P96. Besides regulating glycolytic metabolism, PGK1 mediates other cellular processes, including DNA replication.^42,43^ High mRNA expression of PGK1 is found to be positively associated with poor survival in breast cancer and other cancers.^43^ In addition, PGK1 can promote cell proliferation and inhibit apoptosis in breast cancer.^44,45^ Interestingly, we found that the global protein abundance of NUP153 was overexpressed in P96 compared to P97 (Table S7), suggesting the elevated ubiquitination related to the overall global expression. In contrast, PGK1 showed no difference between the two breast cancer subtypes at the global level, highlighting the importance of studying ubiquitination (Table S7). Additionally, we found that a phosphopeptide of NUP153 was also elevated in P96 compared to P97 (Table S8). Although further examination of the biological significance of differentially expressed ubiquitinated peptides, their corresponding proteins, and the enriched pathways would be required, however, these results indicated the successful application of AUTO-SP for ubiquitinated peptide analysis and its potential for analyzing large-scale clinical samples.

## Conclusions

Sample preparation is one of the critical components to the success of a study. To generate consistent and reproducible data while supporting high sample throughput, we established the AUTO-SP, which adapted some of the essential steps in the 2018 CPTAC protocol by converting them into automated procedures for deep-scale MS-based quantitative proteomic and PTM analyses. In this study, we demonstrated that AUTO-SP produced highly reproducible and consistent results among the PDX samples from the same subtypes, while it was capable of simultaneously handling up to 96 samples. Using the AUTO-SP, we were able to identify more than 10,000 proteins and 25,000 phosphopeptides from the PDX tumor tissues with reproducible results comparable to the 2018 CPTAC protocol.^27^ Moreover, there were notable significant expression differences of the ubiquitinated peptides between the two subtypes as well as on the global protein and phosphopeptide levels. Using the proteins of differentially expressed ubiquitinated peptides, we enriched different pathways. Although we used global peptides to enrich phosphopeptides and ubiquitinated peptides separately in the current study, however, serial enrichment using flow-through from one PTM enrichment for another PTM enrichment is applicable on the AUTO-SP. Co-enrichment of several PTMs (i.e., mixing the enrichment beads for different PTMs together) is another potential approach when the materials are limited. Furthermore, our automated protocols are designed in a way that allows customization to fit users’ specific needs. For instance, users can change the number of incubation cycles when performing PTM enrichment. In addition, additional sample-preparation protocols can be readily developed and integrated into AUTO-SP, extending its capabilities beyond those reported here. In conclusion, AUTO-SP supports high-throughput and reliable automated sample preparation for MS-based proteomic and protein modification analyses of clinical samples.

## Supplementary Material





## Data Availability

Raw data files and search results can be accessed at ProteomeXchange with identifier PXD064088. Search results can also be found in the Supporting Information.

## Notes

Protein digestion was performed using AUTO-SP related to Figure 3 (Figure S1); phosphopeptide enrichment using magnetic Fe-NTA beads on AUTO-SP related to Figure 4 (Figure S2); quantification stability of ubiquitinated peptides that were enriched using the AUTO-SP related to Figure 5 (Figure S3) (PDF)
Expression matrix of global proteins (Table S1); Spearman correlation between manual procedure and AUTO-SP for tryptic digestion related to Figure S1 (Table S2); expression matrix of phosphopeptides (Table S3); Spearman correlation between manual procedure and AUTO-SP for phosphopeptide enrichment using magnetic Fe-NTA beads related to Figure S2 (Table S4); expression matrix of ubiquitinated peptides (Table S5); differential analysis of ubiquitinated peptides (Table S6); differential analysis of global proteins (Table S7); differential analysis of phosphopeptides (Table S8) (XLSX)
