# Supplementary material for: AUTO-SP: Automated Sample Preparation for Analyzing Proteins and Protein Modifications
Source: Anal Chem. 2025 Jul 28;97(31):16751–8. doi: 10.1021/acs.analchem.5c00886 (PMC12355475; doi:10.1021/acs.analchem.5c00886)
Supplement: Supplementary file 1 [file ac5c00886_si_001.pdf]

Supporting Information

AUTO-SP: automated sample preparation for analyzing proteins and protein modifications

T. Mamie Lih[1,*], Liyuan Jiao[1], Lijun Chen[1], Jongmin Woo[1], Yuefan Wang[1], Hui Zhang[1,2,3]

1 Department of Pathology, Johns Hopkins University School of Medicine, Baltimore, Maryland 21231, United States.
2 Department of Oncology, Sidney Kimmel Cancer Center at Johns Hopkins Medical Institutions, Baltimore, Maryland 21231, United States.
3 Department of Urology, Johns Hopkins University School of Medicine, Baltimore, Maryland 21231, United States.

Table of Contents

Supplementary Figures

Figure S1 Protein digestion was performed using AUTO-SP related to Figure 3………… S-2

Figure S2 Phosphopeptide enrichment using magnetic Fe-NTA beads on AUTO-SP related to Figure 4……………………………………………………………………………………… S-3

Figure S3 Quantification stability of ubiquitinated peptides that were enriched using the AUTO-SP related to Figure 5.……………………………………………………………………….. S-4

AUTO-SP protocols

AUTO-SP protocol #1: BCA analysis……………………………………………………………… S-5

AUTO-SP protocol #2: in-solution protein digestion……………………………………….. S-14

AUTO-SP protocol #3: Magnetic bead-based PTM enrichment…………………………….. S-22

Supplementary Tables S1-8 are in a separate XLSX file

Table S1 Expression matrix of global proteins; **Table S2** Spearman correlation between manual procedure and AUTO-SP for tryptic digestion. Related to Figure S1; **Table S3** Expression matrix of phosphopeptides; **Table S4** Spearman correlation between manual procedure and AUTO-SP for phosphopeptide enrichment using magnetic Fe-NTA beads. Related to Figure S2; **Table S5** Expression matrix of ubiquitinated peptides; **Table S6** Differential analysis of ubiquitinated peptides; **Table S7** Differential analysis of global proteins; **Table S8** Differential analysis of phosphopeptides.

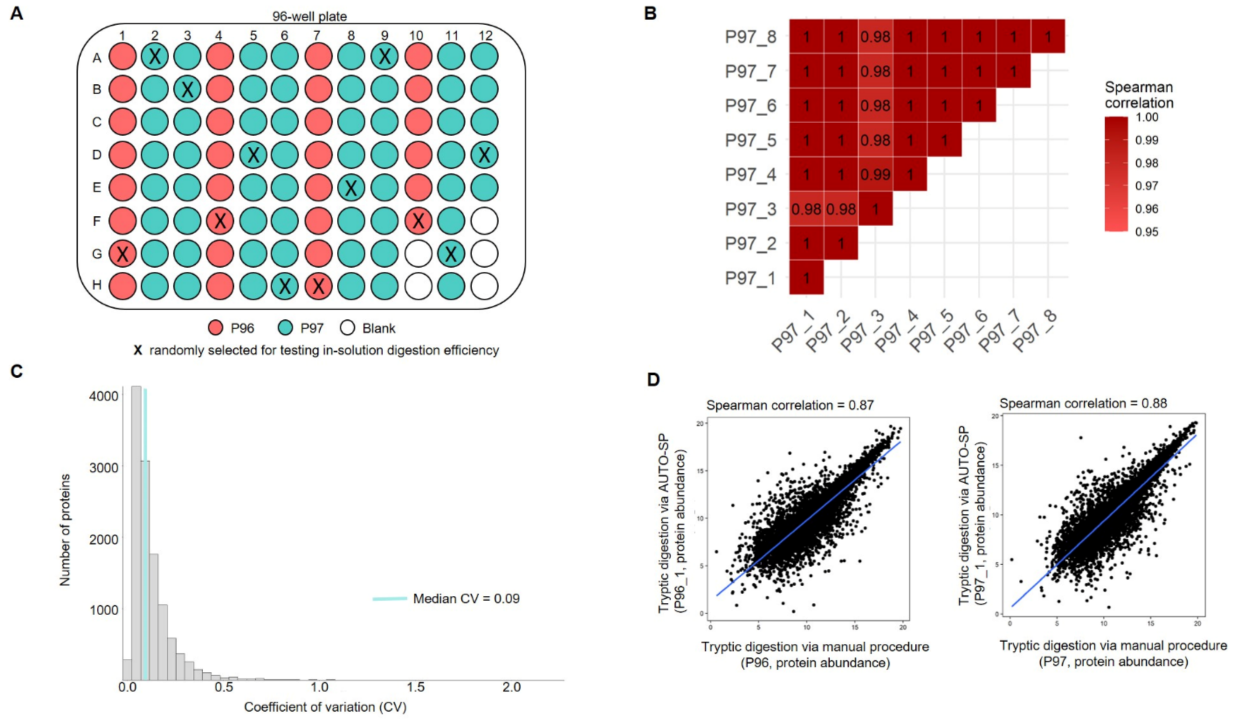

Figure S1. Protein digestion was performed using AUTO-SP related to Figure 3. **A.** Sample layout on a 96-well plate on the AUTO-SP for protein digestion. **B.** Reproducibility of protein digestion on AUTO-SP for P97. **C.** CV of proteins identified in the P97 samples. **D.** A linear relationship is observed between AUTO-SP and manual procedure for tryptic digestion. One replicate from each PDX model is plotted for demonstration purpose.

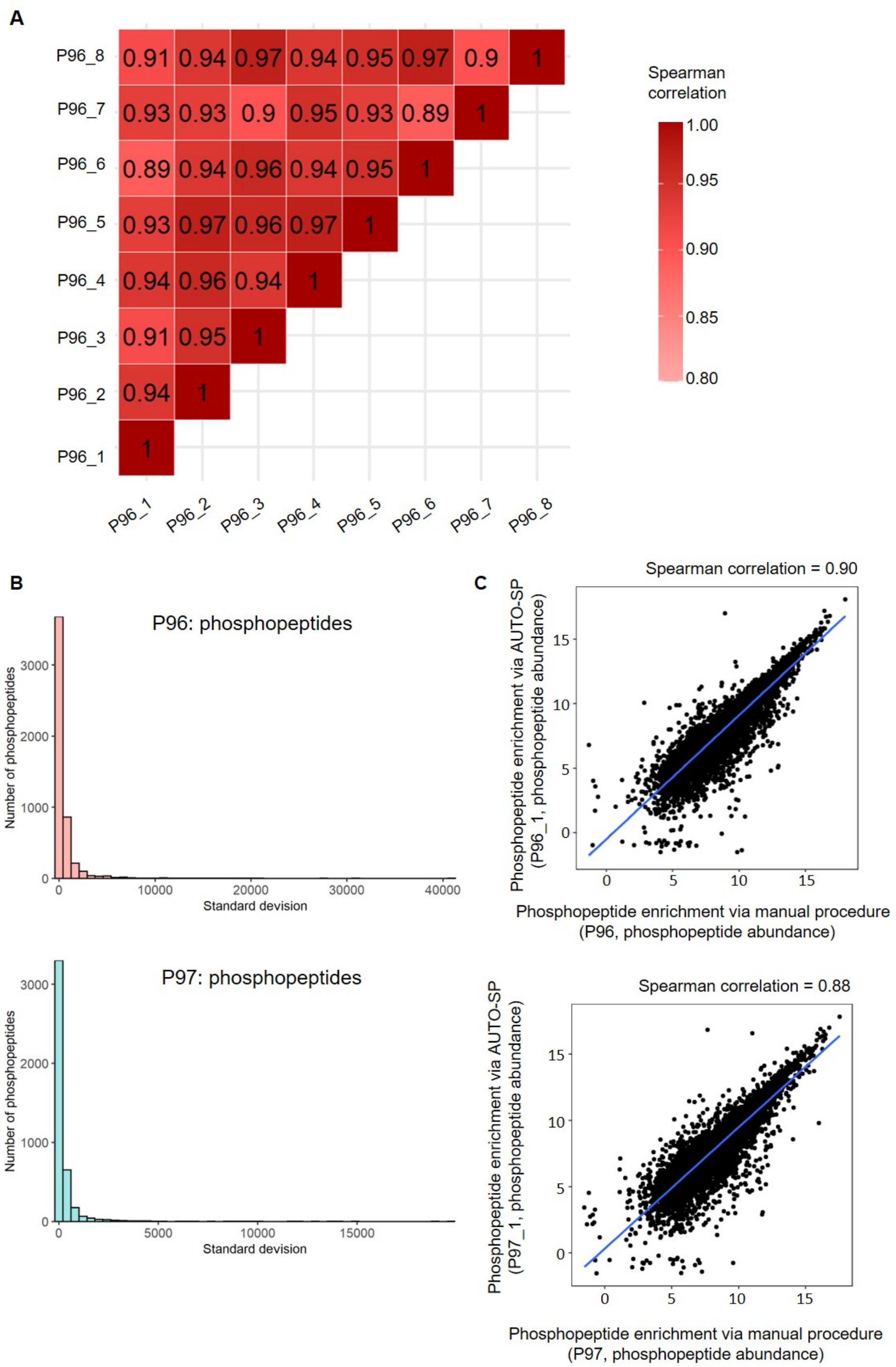

Figure S2. Phosphopeptide enrichment using magnetic Fe-NTA beads on AUTO-SP related to Figure 4. **A.** Reproducibility of phosphopeptide enrichment on AUTO-SP for P96. **B.** Standard deviation computed based on the abundances of phosphopeptides in P96 and P97 showing quantification stability of the enriched data. **C.** The scatter plots show a linear association between manual and automated procedures for phosphopeptide enrichment using magnetic beads. One replicate from each PDX model is plotted for demonstration purpose.

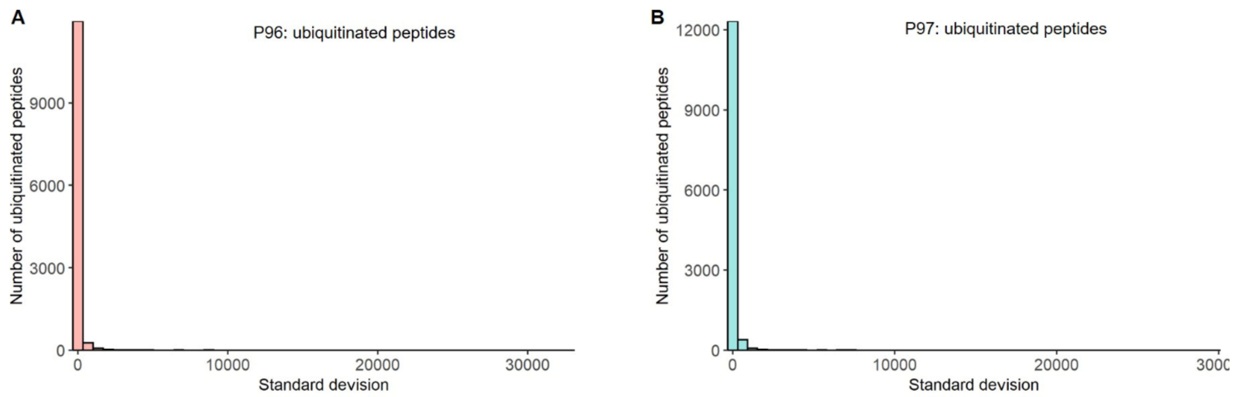

Figure S3. Quantification stability of ubiquitinated peptides that were enriched using the AUTO-SP related to Figure 5. **A.** P96 samples and **B.** P97 samples.

AUTO-SP protocol #1: BCA analysis (copy and paste the following code to a Python script and execute in the Opentrons software)

The protocol is written and executed on Opentrons software v6.3.1 and OT-2 for this study. Adjustments on labware, tip depth etc. may require if executed in other versions or using different labware or tips. The defined parameters can be adjusted according to users' specific needs.

```
def get_values(*names):
    import json
    _all_values = json.loads("""{"std_source_col":1,
    "labware_hs":"corning_96_wellplate_360uL_flat",
    "labware_source":"nest_96_wellplate_100ul_pcr_full_skirt", "src_col_start":2,
    "labware_source2":"nest_12_reservoir_15ml", "src2_col_start":1,
    "tfer_vol":10,"tfer_vol2":200,
    "pip1":"p20_multi_gen2", "pip_side1":"right", "pip_rack1":2, "pip_rack1_start":1,
    "pip2":"p300_multi_gen2","pip_side2":"left", "pip_rack2":5, "pip_rack2_start":1,
    "samples":3,
    "source_loc":3, "source_loc2":8, "num_total_plate":3, "use_new_tip":1,
    "incub_time_in_min":30, "hs_rpm":500, "hs_temp": 37}""")
    return [_all_values[n] for n in names]

#use_new_tip (change tips): 0 = No, 1 = Yes
#hs_temp is in degree Celsius
#sample: number of cols will be transferred from the labware_source to labware_hs; 3
indicates 3 columns (3*8=24 wells), each well with 3 replicates
#the 3rd col on the labware_hs is alway left as blank (1*8 = 8 wells are blank)

import time

metadata = {
    'protocolName': '''BCA_analysis''',
```

```
    'author': 'T. Mamie Lih',
    'apiLevel': '2.14'
}


def run(ctx):

    # get parameter values from json above
    [std_source_col, labware_hs, labware_source, src_col_start, labware_source2,
src2_col_start,
     tfer_vol, tfer_vol2, pip1, pip_side1, pip_rack1, pip_rack1_start, pip2, pip_side2,
pip_rack2, pip_rack2_start, samples,
     source_loc,source_loc2, num_total_plate, use_new_tip, incub_time_in_min, hs_rpm,
hs_temp] = get_values(  # noqa: F821
      'std_source_col', 'labware_hs',
      'labware_source', 'src_col_start', 'labware_source2', 'src2_col_start', 'tfer_vol',
'tfer_vol2', 'pip1', 'pip_side1', 'pip_rack1', 'pip_rack1_start', 'pip2',
       'pip_side2', 'pip_rack2', 'pip_rack2_start', 'samples','source_loc','source_loc2',
       'num_total_plate', 'use_new_tip', 'incub_time_in_min', 'hs_rpm', 'hs_temp')

    ctx.set_rail_lights(False)


    # selected pipette, corresponding tips in defined slot
    tipmap = {'p20_multi_gen2': 'opentrons_96_tiprack_20ul',
            'p20_single_gen2': 'opentrons_96_tiprack_20ul',
            'p300_multi_gen2': 'opentrons_96_tiprack_300ul',
            'p300_single_gen2': 'opentrons_96_tiprack_300ul',
            'p1000_single_gen2': 'opentrons_96_tiprack_1000ul'
            }
```

```
    # define the how deep the tip should go from top of the well
    # these numbers will need to be adjusted based on your OT-2, e.g., labeware
position, offset etc.
    labware_tip_top_well_depth = {'nest_96_wellplate_100ul_pcr_full_skirt': -14.5,
                                  'nest_12_reservoir_15ml': -40.5,
                                  'nest_1_reservoir_195ml': 1,
                                  'nest_96_wellplate_2ml_deep': -37.7,
                                  'corning_96_wellplate_360uL_flat': -14.5,
                                  }

    # set pip for 20 uL
    tip_rack_1 = ctx.load_labware(tipmap.get(pip1), pip_rack1)
    pipette = ctx.load_instrument(pip1, pip_side1, tip_racks=[tip_rack_1])
    pipette.flow_rate.dispense = 4
    pipette.flow_rate.blow_out = 7

    # set pip for 300 uL
    tip_rack_2 = ctx.load_labware(tipmap.get(pip2), pip_rack2)
    pipette2 = ctx.load_instrument(pip2, pip_side2, tip_racks=[tip_rack_2])
    pipette2.flow_rate.blow_out = 100



    # heater shaker module in slot 10, selected plate
    hs_mod = ctx.load_module('heaterShakerModuleV1', '10')

    hs_plate = hs_mod.load_labware(labware_hs, 'BCAplate')

    # open and then close the latch
    hs_mod.open_labware_latch()
    ctx.comment(" latch status {}".format(hs_mod.labware_latch_status))
```

```
# define source labware
source = ctx.load_labware(labware_source, str(source_loc), 'source')
source2 = ctx.load_labware(labware_source2, str(source_loc2), 'BCA')

def change_tipRack_func(tip_col, incr):

    if tip_col > 12:
        tip_col = 1
        ctx.pause('''Place a new tip rack, and then click Resume''')
    else:
        if incr == 1:
            tip_col = tip_col + 1

            if tip_col > 12:
                tip_col = 1
                ctx.pause('''Place a new tip rack, and then click Resume''')

    return tip_col



def shaker_func(hs_speed, incub_time_min, sk_temp):

    # shaking with heat

    hs_mod.set_target_temperature(celsius=sk_temp)

    hs_mod.set_and_wait_for_shake_speed(rpm=hs_speed)
    ctx.delay(seconds=1)
    ctx.comment(" current speed {} ".format(hs_mod.current_speed))
```

```
    # get shaking start time
    start = time.time()

    # wait until the shake time has elapsed (convert min to sec)

    if not ctx.is_simulating():
        while time.time() - start < (incub_time_min * 60):
            continue

    # stop shaking
    hs_mod.deactivate_shaker()
    hs_mod.deactivate_heater()

#region BCA analysis

for i in range(num_total_plate):

    source1_depth = labware_tip_top_well_depth[labware_source]
    source2_depth = labware_tip_top_well_depth[labware_source2]
    dest_depth = labware_tip_top_well_depth[labware_hs]

    std_source_pos = "A" + str(std_source_col)

    #region transfer std (2 cols)

    hs_mod.close_labware_latch()

    for j in range(2):
        dest_well = "A" + str((j + 1))

        tiprack_pos = "A" + str(pip_rack1_start)
```

```
    pipette.pick_up_tip(tip_rack_1[tiprack_pos])

    pipette.mix(1, 15, source[std_source_pos].top(z=source1_depth))
    pipette.aspirate(tfer_vol, source[std_source_pos].top(z=source1_depth))
    pipette.touch_tip()
    pipette.dispense(tfer_vol, hs_plate[dest_well].top(z=dest_depth))
    pipette.blow_out(hs_plate[dest_well].top(z=-6))

    pipette.touch_tip(hs_plate[dest_well])
    pipette.touch_tip(hs_plate[dest_well])

    if use_new_tip == 1:
        pipette.drop_tip()
    else:
        pipette.return_tip()


    pip_rack1_start = change_tipRack_func(pip_rack1_start, 1)

#endregion

#region transfer sample (3 cols)

sample_dest_pos = 4 #leave col 3 (A3 to H3) as blank on the BCA assay plate

for s in range(samples):
    for j in range(3):
        sample_number = s + src_col_start
        source_well_location = "A" + str(sample_number)
        tiprack_pos = "A" + str(pip_rack1_start)
        dest_well = "A" + str(sample_dest_pos)
```

```
        pipette.pick_up_tip(tip_rack_1[tiprack_pos])
        pipette.mix(1, 15, source[source_well_location].top(z=dest_depth))
        pipette.aspirate(tfer_vol, source[source_well_location].top(z=dest_depth))
        pipette.touch_tip()

        pipette.dispense(tfer_vol, hs_plate[dest_well].top(z=dest_depth))
        pipette.blow_out(hs_plate[dest_well].top(z=-6))

        pipette.touch_tip(hs_plate[dest_well])
        pipette.touch_tip(hs_plate[dest_well])

        if use_new_tip == 1:
            pipette.drop_tip()
        else:
            pipette.return_tip()


        sample_dest_pos = sample_dest_pos + 1

        pip_rack1_start = change_tipRack_func(pip_rack1_start, 1)


    #endregion

    #region add BCA

    hs_mod.open_labware_latch()

    ctx.pause('''Place BCA reagent, and click Resume''')
```

```
    hs_mod.close_labware_latch()

    dest_well_start_pos = 1
    tiprack_pos = "A" + str(pip_rack2_start)
    pipette2.pick_up_tip(tip_rack_2[tiprack_pos])

    for s in range(2):
        source_number = s + src2_col_start
        source_column = "A" + str(source_number)

        for j in range(6):

            if s == 0:
                if j == 2:
                    dest_well_start_pos = dest_well_start_pos + 1
                    continue

            dest_well_pos = "A" + str(dest_well_start_pos)

            pipette2.transfer(tfer_vol2, source2[source_column].top(z=source2_depth),
hs_plate[dest_well_pos].top(z=-1),
                              new_tip='never', blow_out=True, blowout_location="destination
well")

            dest_well_start_pos = dest_well_start_pos + 1

    if use_new_tip == 1:
        pipette2.drop_tip()
    else:
        pipette2.return_tip()
```

```
    pip_rack2_start = change_tipRack_func(pip_rack2_start, 1)

    #endregion

    ctx.pause('''Cover the plate, and click Resume''')

    shaker_func(hs_rpm, incub_time_in_min, hs_temp)

    hs_mod.open_labware_latch()

    if i < (num_total_plate-1) :
        ctx.pause('''Click Resume to start next plate''')



    src_col_start = src_col_start + samples
#endregion

ctx.comment('''Process Complete.''')
```

AUTO-SP protocol #2: in-solution protein digestion (copy and paste the following code to a Python script and execute in the Opentrons software)

The protocol is written and executed on Opentrons software v6.3.1 and OT-2 for this study. Adjustments on labware, tip depth etc. may require if executed in other versions or using different labware or tips. The defined parameters can be adjusted according to users' specific needs.

```
def get_values(*names):
    import json
    _all_values =
json.loads("""{"labware_source":"nest_96_wellplate_100ul_pcr_full_skirt",
"source_loc":3,
    "labware_source2":"nest_12_reservoir_15ml", "source_loc2":8,
    "labware_dest":"nest_96_wellplate_2ml_deep",
    "pip1":"p20_multi_gen2", "pip_side1":"right", "pip_rack1":2, "pip_rack1_start":1,
    "pip2":"p300_multi_gen2", "pip_side2":"left", "pip_rack2":5, "pip_rack2_start":1,
    "dtt_vol":0.4, "iaa_vol":0.75, "res_vol_dil":43, "res_vol_lysc":2.4, "res_vol_tryp":4.8,
"res_vol_acid":3, "res_vol_acid2":8,
    "dtt_start":1, "iaa_start":2, "dil_res_start":1, "lysc_res_start":3, "tryp_res_start":5,
"acid_res_start":6, "acid2_res_start":8,
    "tfer_dtt":5, "tfer_iaa":10, "tfer_lysc":32, "tfer_tryp":64, "tfer_dil":588, "tfer_acid":36,
"tfer_acid2":100,
    "dtt_incub_time":0.5, "iaa_incub_time":45, "lysc_incub_time":120,
"tryp_incub_time":960, "acid_incub_time":1, "hs_rpm":500,
    "dest_well_total":96, "dest_well_eachCol": 8, "dest_cols": "1, 2, 3, 4, 5, 6, 7, 8, 9",
    "use_new_tip":1, "mix_num_after_tfer_to_dest":3, "dilute_with_01_FA":1}""")
    return [_all_values[n] for n in names]

#dtt_vol, iaa_vol, res_vol_dil, res_vol_lysc, res_vol_tryp, res_vol_acid, in mL (this is
total volumn)
#incubation time is in min
#use_new_tip (change tips): 0 = No, 1 = Yes
#dilute_with_01_FA: whether to further dilute using 0.1% FA, 0 = No, 1 = Yes

import time

metadata = {
    'protocolName': '''protein_digestion_DTT_HS''',
    'author': 'T. Mamie Lih',
    'apiLevel': '2.14'
}


def run(ctx):
    # get parameter values from json above
    [labware_source, source_loc, labware_source2, source_loc2, labware_dest,
     pip1, pip_side1, pip_rack1, pip_rack1_start, pip2, pip_side2, pip_rack2,
```

```
pip_rack2_start,
     dtt_start, iaa_start, dil_res_start, lysc_res_start, tryp_res_start,
acid_res_start, acid2_res_start,
     tfer_dtt, tfer_iaa, tfer_lysc, tfer_tryp, tfer_dil, tfer_acid,
tfer_acid2,
     dtt_incub_time, iaa_incub_time, lysc_incub_time, tryp_incub_time,
acid_incub_time, hs_rpm,
     dest_well_total, dest_well_eachCol, dest_cols, use_new_tip,
mix_num_after_tfer_to_dest, dilute_with_01_FA] = get_values(
        'labware_source', 'source_loc', 'labware_source2', 'source_loc2',
'labware_dest',
        'pip1', 'pip_side1', 'pip_rack1', 'pip_rack1_start', 'pip2',
'pip_side2', 'pip_rack2', 'pip_rack2_start',
        'dtt_start', 'iaa_start', 'dil_res_start', 'lysc_res_start',
'tryp_res_start', 'acid_res_start', 'acid2_res_start',
        'tfer_dtt', 'tfer_iaa', 'tfer_lysc', 'tfer_tryp', 'tfer_dil',
'tfer_acid', 'tfer_acid2',
        'dtt_incub_time', 'iaa_incub_time', 'lysc_incub_time',
'tryp_incub_time', 'acid_incub_time', 'hs_rpm',
        'dest_well_total', 'dest_well_eachCol', 'dest_cols', 'use_new_tip',
'mix_num_after_tfer_to_dest', 'dilute_with_01_FA'
    )

    ctx.set_rail_lights(False)

    # define how deep the tip should go from top of the well
    # these numbers will need to be adjusted based on your OT-2, e.g.,
labeware position, offset etc.
    labware_tip_top_well_depth = {'nest_96_wellplate_100ul_pcr_full_skirt': -
15.4,
                                  'nest_12_reservoir_15ml': -41.2,
                                  'nest_1_reservoir_195ml': 1,
                                  'nest_96_wellplate_2ml_deep': -37.7
                                  }

    # selected pipette, corresponding tips in defined slot
    tipmap = {'p20_multi_gen2': 'opentrons_96_tiprack_20ul',
              'p20_single_gen2': 'opentrons_96_tiprack_20ul',
              'p300_multi_gen2': 'opentrons_96_tiprack_300ul',
              'p300_single_gen2': 'opentrons_96_tiprack_300ul',
              'p1000_single_gen2': 'opentrons_96_tiprack_1000ul'
              }

    # define total vol can be held in a channel of the reservoir and a col (8
wells) of a 96-well plate (PCR)
    labware_total_vol_hold = {'nest_12_reservoir_15ml': 15,
                              'nest_96_wellplate_100ul_pcr_full_skirt':1.3}

    # set pip for 20 uL multi-channel

    tip_rack_1 = ctx.load_labware(tipmap.get(pip1), pip_rack1)
    pipette = ctx.load_instrument(pip1, pip_side1, tip_racks=[tip_rack_1])
    pipette.flow_rate.aspirate = 5
    pipette.flow_rate.dispense = 20
    pipette.flow_rate.blow_out = 25

    # set pip for 300 uL multi-channel
```

```
    tip_rack_2 = ctx.load_labware(tipmap.get(pip2), pip_rack2)
    pipette2 = ctx.load_instrument(pip2, pip_side2, tip_racks=[tip_rack_2])
    pipette2.flow_rate.blow_out = 350


    # define source labware
    source = ctx.load_labware(labware_source, str(source_loc), 'sol1_plate')

    source2 = ctx.load_labware(labware_source2, str(source_loc2),
'sol2_plate')

    # define heater shaker module
    hs_mod = ctx.load_module('heaterShakerModuleV1', '10')

    # open the latch
    hs_mod.open_labware_latch()
    ctx.comment(" latch status {}".format(hs_mod.labware_latch_status))

    # define dest labware
    dest = hs_mod.load_labware(labware_dest, 'proteins_plate')
    dest_cols_array = dest_cols.split(", ")

    def change_tipRack_func(tip_col, incr):

        if incr == 1:
            tip_col = tip_col + 1

        if tip_col > 12:
            tip_col = 1
            ctx.pause('''Place a new tip rack, and then click Resume''')

        return tip_col

    # transfer DTT and IAA function

    def transfer_smallVol_20ul(source_plate_labware, source_plate,
source_start, rack_start, dest_plate_labware, dest_plate, tfer_vol):

        source_depth = labware_tip_top_well_depth[source_plate_labware]
        dest_depth = labware_tip_top_well_depth[dest_plate_labware]

        each_draw_buf_vol_ml = (tfer_vol / 1000) * dest_well_eachCol
        change_buf_channel_every_n_iteration =
int(labware_total_vol_hold[source_plate_labware] / each_draw_buf_vol_ml)

        buf_channel_incr = source_start
        buf_pos = "A" + str(buf_channel_incr)

        count_buf_incr = 1

        for d in dest_cols_array:
            tiprack_pos = "A" + str(rack_start)
            pipette.pick_up_tip(tip_rack_1[tiprack_pos])

            dest_well = "A" + d

            # determine if pipette2 should aspirate buf from a new channel
```

```
            if count_buf_incr > change_buf_channel_every_n_iteration:
                count_buf_incr = 1
                buf_channel_incr = buf_channel_incr + 1
                buf_pos = "A" + str(buf_channel_incr)

            pipette.transfer(tfer_vol,
source_plate[buf_pos].top(z=source_depth),
                             dest_plate[dest_well].top(z=dest_depth),
                             new_tip='never',
mix_after=(mix_num_after_tfer_to_dest, 20))

            pipette.blow_out(dest_plate[dest_well].top(z=0.1))

            count_buf_incr = count_buf_incr + 1

            if use_new_tip == 1:
                pipette.drop_tip()
            else:
                pipette.return_tip()

            rack_start = change_tipRack_func(rack_start, 1)


        return rack_start


    # transfer dilution_tris, lysc, trypsin, dilution_FA

    def transfer_largerVol_300ul(source_plate_labware, source_plate,
rack_start, dest_plate_labware, dest_plate, source_start, tfer_vol,
mixRequired):

        source_depth = labware_tip_top_well_depth[source_plate_labware]
        dest_depth = labware_tip_top_well_depth[dest_plate_labware]

        each_draw_buf_vol_ml = (tfer_vol / 1000) * dest_well_eachCol
        change_buf_channel_every_n_iteration =
int(labware_total_vol_hold[source_plate_labware] / each_draw_buf_vol_ml)

        buf_channel_incr = source_start
        buf_pos = "A" + str(buf_channel_incr)

        count_buf_incr = 1

        tiprack_pos = "A" + str(rack_start)
        pipette2.pick_up_tip(tip_rack_2[tiprack_pos])

        last_col = dest_cols_array[-1]

        for d in dest_cols_array:

            dest_well = "A" + d

            # determine if pipette2 should aspirate buf from a new channel
            if count_buf_incr > change_buf_channel_every_n_iteration:
                count_buf_incr = 1
                buf_channel_incr = buf_channel_incr + 1
```

```
                buf_pos = "A" + str(buf_channel_incr)

            if mixRequired == 1:

                if tfer_vol > 300:
                    pipette2.transfer(tfer_vol,
source_plate[buf_pos].top(z=source_depth),
                                      dest_plate[dest_well].top(z=0.1),
new_tip='never',
                                      blow_out=True,
blowout_location="destination well")
                else:
                    pipette2.transfer(tfer_vol,
source_plate[buf_pos].top(z=source_depth),

dest_plate[dest_well].top(z=dest_depth), new_tip='never',
                                      blow_out=False,
blowout_location="destination well")

                pipette2.mix(mix_num_after_tfer_to_dest, 300,
dest_plate[dest_well].top(z=dest_depth))
                pipette2.blow_out(dest_plate[dest_well].top(z=0.1))

                if use_new_tip == 1:
                    pipette2.drop_tip()
                else:
                    pipette2.return_tip()

                rack_start = change_tipRack_func(rack_start, 1)

                if d != last_col:
                    tiprack_pos = "A" + str(rack_start)
                    pipette2.pick_up_tip(tip_rack_2[tiprack_pos])
            else:
                pipette2.transfer(tfer_vol,
source_plate[buf_pos].top(z=source_depth),
                                  dest_plate[dest_well].top(z=0.1),
new_tip='never',
                                  blow_out=True,
blowout_location="destination well")

            count_buf_incr = count_buf_incr + 1

        if mixRequired == 0:
            if use_new_tip == 1:
                pipette2.drop_tip()
            else:
                pipette2.return_tip()
            rack_start = change_tipRack_func(rack_start, 1)

        return rack_start

    def shaker_func(hs_speed, incub_time_min, withHeat):

        # shaking without heating

        if withHeat==1:
```

```
            hs_mod.set_target_temperature(celsius=37)

        hs_mod.set_and_wait_for_shake_speed(rpm=hs_speed)
        ctx.delay(seconds=1)
        ctx.comment(" current speed {} ".format(hs_mod.current_speed))

        # get shaking start time
        start = time.time()

        # wait until the shake time has elapsed (convert min to sec)

        if not ctx.is_simulating():
            while time.time() - start < (incub_time_min * 60):
                continue

        # stop shaking
        hs_mod.deactivate_shaker()

        if withHeat==1:
            hs_mod.deactivate_heater()


    # region Step 1: transfer DTT to dest
    tiprack1_start = pip_rack1_start

    hs_mod.close_labware_latch()

    tiprack1_start = transfer_smallVol_20ul(labware_source, source,
dtt_start, tiprack1_start, labware_dest, dest, tfer_dtt)

    hs_mod.open_labware_latch()
    ctx.pause('''Cover the plate and then click Resume to start
incubation.''')
    hs_mod.close_labware_latch()

    shaker_func(hs_rpm, dtt_incub_time, 1)

    hs_mod.open_labware_latch()
    ctx.comment(" latch status {}".format(hs_mod.labware_latch_status))

    #endregion

    ctx.pause('''Add IAA: cover Opentrons to keep out the light, and then
click Resume.''')
    hs_mod.close_labware_latch()

    # region transfer IAA to dest

    tiprack1_start = transfer_smallVol_20ul(labware_source, source,
iaa_start, tiprack1_start, labware_dest, dest, tfer_iaa)

    hs_mod.open_labware_latch()
    ctx.pause('''Cover the plate and then click Resume to start
incubation.''')
    hs_mod.close_labware_latch()

    shaker_func(hs_rpm, iaa_incub_time, 0)
```

```
    # endregion

    hs_mod.open_labware_latch()
    ctx.pause('''Add Tris: remove the covers from Opentrons and the plate,
and then click Resume.''')
    hs_mod.close_labware_latch()

    # region  Dilution 1:4 to decrease Urea concentration below 2M

    tiprack2_start = pip_rack2_start

    tiprack2_start = transfer_largerVol_300ul(labware_source2, source2,
tiprack2_start, labware_dest, dest, dil_res_start, tfer_dil, 0)

    # endregion

    ctx.pause('''Add Lys-C''')

    # region transfer LysC

    tiprack2_start = transfer_largerVol_300ul(labware_source, source,
tiprack2_start, labware_dest, dest, lysc_res_start, tfer_lysc, 1)

    hs_mod.open_labware_latch()
    ctx.pause('''Cover the plate and then click Resume to start
incubation.''')
    hs_mod.close_labware_latch()

    shaker_func(hs_rpm, lysc_incub_time, 0)

    # endregion

    ctx.pause('''Add Trypsin''')

    # region transfer Trypsin
    tiprack2_start = transfer_largerVol_300ul(labware_source, source,
tiprack2_start, labware_dest, dest, tryp_res_start, tfer_tryp, 1)

    hs_mod.open_labware_latch()
    ctx.pause('''Cover the plate and then click Resume to start
incubation.''')
    hs_mod.close_labware_latch()

    shaker_func(hs_rpm, tryp_incub_time, 0)

    # endregion

    ctx.pause('''Add 50% FA.''')

    # region transfer 50% FA

    tiprack2_start = transfer_largerVol_300ul(labware_source2, source2,
tiprack2_start, labware_dest, dest,acid_res_start, tfer_acid, 1)

    # endregion
```

```
    # region transfer 0.1% FA
    if dilute_with_01_FA == 1:
        ctx.pause('''Add 0.1% FA.''')
        tiprack2_start = transfer_largerVol_300ul(labware_source2, source2,
tiprack2_start, labware_dest, dest,
                                                  acid2_res_start,
tfer_acid2, 1)

    # endregion

    hs_mod.open_labware_latch()
    ctx.pause('''Cover the plate and then click Resume.''')
    hs_mod.close_labware_latch()

    shaker_func(hs_rpm, acid_incub_time, 0)

    hs_mod.open_labware_latch()

    ctx.comment('''Process Complete.''')
```

AUTO-SP protocol #3: Magnetic bead-based PTM enrichment (copy and paste the following code to a Python script and execute in the Opentrons software)

The protocol is written and executed on Opentrons software v6.3.1 and OT-2 for this study. Adjustments on labware, tip depth etc. may require if executed in other versions or using different labware or tips. The defined parameters can be adjusted according to users' specific needs.

```
def get_values(*names):
    import json
    _all_values = json.loads("""{"pip":"p300_multi_gen2", "pip_side":"left",
"rack300_slot":5, "rack300_start_col":0,
    "source_buf_labware":"nest_12_reservoir_15ml", "source_buf_labware_slot":8,
    "source_buf_AfterBind_start_col":1, "source_buf2_AfterBind_start_col":3,
"source_elute_start_col":6, "source_collect_bead_start_col":12,
    "source_pep_flow_labware":"thermoscientificnunc_96_wellplate_1300ul",
"source_pep_labware_slot":3,
    "dest_mag_labware":"nest_96_wellplate_100ul_pcr_full_skirt", "dest_mag_cols":"1,
2, 3, 4, 5, 6, 7, 8, 9, 10, 11, 12",
    "dest_elute_labware":"thermoscientificnunc_96_wellplate_1300ul",
"dest_elute_labware_slot":4,
    "waste_labware":"nest_1_reservoir_195ml", "waste_labware_slot":6,
    "beads_aliquot_tfer_vol_ul":150,
    "wash_buff_each_channel_ml":15, "wash_buff_each_channel_ml_2":18,
"beads_wash_tfer_vol_ul":150, "air_gap_ul":5,
    "pep_mag_tfer_vol_ul":150, "beads_bind_wash_cycle":3,
"beads_bind_wash_cycle_2":0,
    "elute_buff_each_channel_ml":15, "elute_tfer_vol_ul":100, "elute_cycle":3,
"elute_final_mix_cycle":8,
    "collect_bead_tfer_vol_ul":50,
    "mix_mag_cycle":10, "mix_mag_elu_cycle":15, "mix_mag_activate_cycle":1,
"mix_mag_activate_cycle_2":1, "mix_mag_brief_elu_cycle":5,
```

```
    "pause_pip_sec":15, "pause_pip_sec_tipWash":5, "pause_pip_sec_asp":2,
    "magnet_height":6.4, "pause_btw_sec":0, "pause_btw_elu_sec":0, "mix_set_vol":130,
    "incubation_mix_mag_cycle":3, "mix_max_bead_bind_wash_cycle": 5,
    "sample_each_col":8,
    "use_new_tip":1}""")
    return [_all_values[n] for n in names]

import time

metadata = {
    'protocolName': '''Magnetic_bead_PTM_enrichment''',
    'author': 'T. Mamie Lih',
    'apiLevel': '2.14'
}


def run(ctx):
    # region get parameter values from json above
    [pip, pip_side, rack300_slot, rack300_start_col,
     source_buf_labware, source_buf_labware_slot,
     source_buf_AfterBind_start_col, source_buf2_AfterBind_start_col,
source_elute_start_col,
     source_collect_bead_start_col,
     source_pep_flow_labware, source_pep_labware_slot,
     dest_mag_labware, dest_mag_cols,
     dest_elute_labware, dest_elute_labware_slot,
     waste_labware, waste_labware_slot,
     beads_aliquot_tfer_vol_ul,
     wash_buff_each_channel_ml, wash_buff_each_channel_ml_2,
beads_wash_tfer_vol_ul, air_gap_ul,
     pep_mag_tfer_vol_ul, beads_bind_wash_cycle, beads_bind_wash_cycle_2,
```

```
    elute_buff_each_channel_ml, elute_tfer_vol_ul, elute_cycle, elute_final_mix_cycle,
    collect_bead_tfer_vol_ul,
    mix_mag_cycle, mix_mag_elu_cycle, mix_mag_activate_cycle,
mix_mag_activate_cycle_2, mix_mag_brief_elu_cycle,
    pause_pip_sec, pause_pip_sec_tipWash, pause_pip_sec_asp,
    magnet_height, pause_btw_sec, pause_btw_elu_sec, mix_set_vol,
    incubation_mix_mag_cycle, mix_max_bead_bind_wash_cycle,
    sample_each_col,
    use_new_tip] = get_values(
        'pip', 'pip_side', 'rack300_slot', 'rack300_start_col',
        'source_buf_labware', 'source_buf_labware_slot',
        'source_buf_AfterBind_start_col', 'source_buf2_AfterBind_start_col',
'source_elute_start_col',
        'source_collect_bead_start_col',
        'source_pep_flow_labware', 'source_pep_labware_slot',
        'dest_mag_labware', 'dest_mag_cols',
        'dest_elute_labware', 'dest_elute_labware_slot',
        'waste_labware', 'waste_labware_slot',
        'beads_aliquot_tfer_vol_ul',
        'wash_buff_each_channel_ml', 'wash_buff_each_channel_ml_2',
'beads_wash_tfer_vol_ul', 'air_gap_ul',
        'pep_mag_tfer_vol_ul', 'beads_bind_wash_cycle', 'beads_bind_wash_cycle_2',
        'elute_buff_each_channel_ml', 'elute_tfer_vol_ul', 'elute_cycle',
'elute_final_mix_cycle',
        'collect_bead_tfer_vol_ul',
        'mix_mag_cycle', 'mix_mag_elu_cycle', 'mix_mag_activate_cycle',
'mix_mag_activate_cycle_2',
        'mix_mag_brief_elu_cycle',
        'pause_pip_sec', 'pause_pip_sec_tipWash', 'pause_pip_sec_asp',
        'magnet_height', 'pause_btw_sec', 'pause_btw_elu_sec', 'mix_set_vol',
        'incubation_mix_mag_cycle', 'mix_max_bead_bind_wash_cycle',
```

```
    'sample_each_col',
    'use_new_tip')
# endregion

ctx.set_rail_lights(False)  # not turning on the lights

# region set pipettes and tip racks
tipmap = {'p20_multi_gen2': 'opentrons_96_tiprack_20ul',
         'p20_single_gen2': 'opentrons_96_tiprack_20ul',
         'p300_multi_gen2': 'opentrons_96_tiprack_300ul',
         'p300_single_gen2': 'opentrons_96_tiprack_300ul',
         'p1000_single_gen2': 'opentrons_96_tiprack_1000ul'
         }

# set pip for 300 uL
tip_rack_300 = ctx.load_labware(tipmap.get(pip), rack300_slot)
pip300 = ctx.load_instrument(pip, pip_side, tip_racks=[tip_rack_300])
pip300.flow_rate.blow_out = 300

# endregion

# region set magnetic modules

mag_mod = ctx.load_module('magnetic module gen2', '1')

# endregion

# region set up source plates and dest plates

# define depth for tip can go for each labware
labware_tip_top_well_depth = {'nest_96_wellplate_100ul_pcr_full_skirt': -14.9,
```

```
                    'nest_12_reservoir_15ml': -40.5,
                    'nest_1_reservoir_195ml': 1,
                    'nest_96_wellplate_2ml_deep': -37.7,
                    'opentrons_96_wellplate_200ul_pcr_full_skirt': -16.6,
                    'thermoscientificnunc_96_wellplate_1300ul': -26.5
                    }

    source_buf_plate = ctx.load_labware(source_buf_labware,
str(source_buf_labware_slot), 'beads_buffer')
    source_buf_plate_depth = labware_tip_top_well_depth[source_buf_labware]

    source_pep_flow_plate = ctx.load_labware(source_pep_flow_labware,
str(source_pep_labware_slot), 'pep_flow')
    source_pep_flow_plate_depth =
labware_tip_top_well_depth[source_pep_flow_labware] - 1

    dest_mag_plate = mag_mod.load_labware(dest_mag_labware, 'binding')
    dest_mag_plate_depth = labware_tip_top_well_depth[dest_mag_labware] - 0.38
    dest_mag_plate_depth_mix = labware_tip_top_well_depth[dest_mag_labware] + 0.6

    dest_elute_plate = ctx.load_labware(dest_elute_labware,
str(dest_elute_labware_slot), 'pep_elute')
    dest_elute_plate_depth = labware_tip_top_well_depth[dest_elute_labware]

    waste_plate = ctx.load_labware(waste_labware, str(waste_labware_slot),
'wash_waste')

    dest_mag_cols_array = dest_mag_cols.split(", ")

    prev_elution_channel_incr = 0
```

```
    # endregion

    # region functions

    def change_tipRack_func(tip_col, incr):

        if incr == 1:
            tip_col = tip_col + 1

        if tip_col > 12:
            tip_col = 1
            ctx.pause('''Place a new tip rack, and then click Resume''')

        return tip_col

    def mix_func(pip_mix_cycles, mix_vol, mix_labware, mix_labware_wells_pos,
mix_labware_tip_depth,
                 mix_labware_tip_depth2, mix_step1, to_waste, tfer_vol_to_dest,
to_collection, coll_labware, coll_labware_wells_pos, coll_labware_tip_depth,
Is_tip_wash):

        if mix_step1 == 1:
            pip300.mix(pip_mix_cycles, mix_vol,
mix_labware[mix_labware_wells_pos].top(z=mix_labware_tip_depth))
            pip300.blow_out(mix_labware[mix_labware_wells_pos].top(z=0.1))

            if Is_tip_wash == 2:
                pip300.mix(mix_mag_activate_cycle, mix_vol,
mix_labware[mix_labware_wells_pos].top(z=mix_labware_tip_depth))
                pip300.blow_out(mix_labware[mix_labware_wells_pos].top(z=0.1))
```

```
    if to_waste == 1:

        # tip wash before remove waste
        mag_mod.engage(height_from_base=magnet_height)
        ctx.delay(seconds=pause_pip_sec)

        if Is_tip_wash == 1:
            pip300.mix(mix_mag_activate_cycle, mix_vol,
mix_labware[mix_labware_wells_pos].top(z=mix_labware_tip_depth))
            pip300.blow_out(mix_labware[mix_labware_wells_pos].top(z=0.1))
            ctx.delay(seconds=pause_pip_sec_tipWash)

        final_tfer_vol = tfer_vol_to_dest + 5.5
        pip300.aspirate(final_tfer_vol,
mix_labware[mix_labware_wells_pos].top(z=mix_labware_tip_depth2))
        ctx.delay(seconds=pause_pip_sec_asp)

        if to_collection == 0:
            pip300.dispense(final_tfer_vol, waste_plate['A1'].top(z=1))
            pip300.blow_out(waste_plate['A1'].top(z=1))
        else:
            pip300.dispense(final_tfer_vol, coll_labware[coll_labware_wells_pos].top(z=-
1))
            pip300.blow_out(coll_labware[coll_labware_wells_pos].top(z=-1))


        mag_mod.disengage()



    # endregion
```

```
def removeWashBuffer_beforeBind_func(tip_col):

    pip300.flow_rate.aspirate = 94
    pip300.flow_rate.dispense = 94

    tip_col = change_tipRack_func(tip_col, 1)
    tip_pos = "A" + str(tip_col)
    pip300.pick_up_tip(tip_rack_300[tip_pos])

    mag_mod.engage(height_from_base=magnet_height)
    ctx.delay(seconds=pause_pip_sec)

    final_tfer_vol = beads_aliquot_tfer_vol_ul + 5.5

    for d in dest_mag_cols_array:
        mag_col = "A" + d

        pip300.aspirate(final_tfer_vol,
dest_mag_plate[mag_col].top(z=dest_mag_plate_depth))
        ctx.delay(seconds=pause_pip_sec_asp)

        pip300.dispense(final_tfer_vol, waste_plate['A1'].top(z=1))
        pip300.blow_out(waste_plate['A1'].top(z=1))

    mag_mod.disengage()

    if use_new_tip == 1:
        pip300.drop_tip()
    else:
        pip300.return_tip()
```

```
    return tip_col

def addPep_func(tip_col):

    pip300.flow_rate.aspirate = 60
    pip300.flow_rate.dispense = 60

    for d in dest_mag_cols_array:
        mag_col = "A" + d

        pep_pos = "A" + d


        tip_col = change_tipRack_func(tip_col, 1)
        tip_pos = "A" + str(tip_col)
        pip300.pick_up_tip(tip_rack_300[tip_pos])

        # region add sample to the 96-well plate on the magnetic module
        pip300.aspirate(pep_mag_tfer_vol_ul,
source_pep_flow_plate[pep_pos].top(z=source_pep_flow_plate_depth))
        ctx.delay(seconds=pause_pip_sec_asp)

        pip300.dispense(pep_mag_tfer_vol_ul, dest_mag_plate[mag_col].top(z=-5))
        pip300.blow_out(dest_mag_plate[mag_col].top(z=-1))

        if use_new_tip == 1:
            pip300.drop_tip()
        else:
            pip300.return_tip()
```

```
    return tip_col

def Pep_incubation_func(tip_col):

    pip300.flow_rate.aspirate = 60
    pip300.flow_rate.dispense = 100

    for d in dest_mag_cols_array:
        mag_col = "A" + d

        tip_col = change_tipRack_func(tip_col, 1)
        tip_pos = "A" + str(tip_col)
        pip300.pick_up_tip(tip_rack_300[tip_pos])

        # start incubation by mixing the sample with beads in the well in defined number
of cycles

        mix_func(mix_mag_cycle, mix_set_vol, dest_mag_plate, mag_col,
dest_mag_plate_depth_mix,
                 dest_mag_plate_depth, 1, 0, 0, 0, "0", 0, 0, 0)
        pip300.blow_out(dest_mag_plate[mag_col].top(z=0.1))

        if use_new_tip == 1:
            pip300.drop_tip()
        else:
            pip300.return_tip()

    return tip_col

def wash_elution_afterBind_func(tip_col, wash1_activate, wash2_activate):
```

```
        # region wash buffer
        each_draw_buf_vol_ml = (beads_wash_tfer_vol_ul / 1000) * sample_each_col
        change_buf_channel_every_n_iteration = int(wash_buff_each_channel_ml /
each_draw_buf_vol_ml)

        buf_channel_incr = source_buf_AfterBind_start_col
        buf_pos = "A" + str(buf_channel_incr)

        count_buf_incr = 1

        # endregion

        # region wash buffer 2

        each_draw_buf2_vol_ml = (beads_wash_tfer_vol_ul / 1000) * sample_each_col
        change_buf2_channel_every_n_iteration = int(wash_buff_each_channel_ml_2 /
each_draw_buf2_vol_ml)

        buf2_channel_incr = source_buf2_AfterBind_start_col
        buf2_pos = "A" + str(buf2_channel_incr)

        count_buf2_incr = 1

        # endregion

        # region elution buffer
        each_draw_elute_vol_ml = (elute_tfer_vol_ul / 1000) * sample_each_col
        change_elute_channel_every_n_iteration = int(elute_buff_each_channel_ml /
each_draw_elute_vol_ml)

        elute_channel_incr = source_elute_start_col
```

```
    elute_pos = "A" + str(elute_channel_incr)

    prev_elution_channel_incr = 1
    # endregion

    # region collect flow through, wash 1, wash2, add elution, one column at a time

    for d in dest_mag_cols_array:
        mag_col = "A" + d
        pep_pos = "A" + d

        mag_mod.engage(height_from_base=magnet_height)
        ctx.delay(seconds=pause_pip_sec)

        # region collect flowthrough
        pip300.flow_rate.aspirate = 60
        pip300.flow_rate.dispense = 60

        tip_col = change_tipRack_func(tip_col, 1)
        tip_pos = "A" + str(tip_col)
        pip300.pick_up_tip(tip_rack_300[tip_pos])

        pip300.aspirate(pep_mag_tfer_vol_ul,
dest_mag_plate[mag_col].top(z=dest_mag_plate_depth))
        ctx.delay(seconds=pause_pip_sec_asp)

        pip300.dispense(pep_mag_tfer_vol_ul,
source_pep_flow_plate[pep_pos].top(z=source_pep_flow_plate_depth))

        pip300.blow_out(source_pep_flow_plate[pep_pos].top(z=0.1))
```

```
if use_new_tip == 1:
    pip300.drop_tip()
else:
    pip300.return_tip()

mag_mod.disengage()
# endregion

pip300.flow_rate.aspirate = 120
pip300.flow_rate.dispense = 120

# region wash 1
if wash1_activate == 1:
    for w in range(beads_bind_wash_cycle):
        tip_col = change_tipRack_func(tip_col, 1)
        tip_pos = "A" + str(tip_col)
        pip300.pick_up_tip(tip_rack_300[tip_pos])

        # draw clean wash buffer #1
        if count_buf_incr > change_buf_channel_every_n_iteration:
            count_buf_incr = 1
            buf_channel_incr = buf_channel_incr + 1
            buf_pos = "A" + str(buf_channel_incr)

        pip300.transfer(beads_wash_tfer_vol_ul,
                        source_buf_plate[buf_pos].top(z=source_buf_plate_depth),
                        dest_mag_plate[mag_col].top(z=-1),
                        new_tip='never', blow_out=False)

        pip300.blow_out(dest_mag_plate[mag_col].top(z=0.1))
```

```
            count_buf_incr = count_buf_incr + 1

            # wash
            mix_func(mix_max_bead_bind_wash_cycle, beads_wash_tfer_vol_ul,
                     dest_mag_plate, mag_col, dest_mag_plate_depth_mix,
dest_mag_plate_depth, 1, 1,
                     beads_wash_tfer_vol_ul, 1,source_pep_flow_plate, pep_pos,
source_pep_flow_plate_depth, 0)

            if use_new_tip == 1:
                pip300.drop_tip()
            else:
                pip300.return_tip()


        # endergion

        # region wash 2

        if wash2_activate == 1:
            for w in range(beads_bind_wash_cycle_2):
                tip_col = change_tipRack_func(tip_col, 1)
                tip_pos = "A" + str(tip_col)
                pip300.pick_up_tip(tip_rack_300[tip_pos])

                # draw clean wash buffer #1
                if count_buf2_incr > change_buf2_channel_every_n_iteration:
                    count_buf2_incr = 1
                    buf2_channel_incr = buf2_channel_incr + 1
                    buf2_pos = "A" + str(buf2_channel_incr)
```

```
            pip300.transfer(beads_wash_tfer_vol_ul,
                            source_buf_plate[buf2_pos].top(z=source_buf_plate_depth),
                            dest_mag_plate[mag_col].top(z=-1),
                            new_tip='never', blow_out=False)

            pip300.blow_out(dest_mag_plate[mag_col].top(z=0.1))

            count_buf2_incr = count_buf2_incr + 1

            # wash
            mix_func(mix_max_bead_bind_wash_cycle, beads_wash_tfer_vol_ul,
                     dest_mag_plate, mag_col, dest_mag_plate_depth_mix,
dest_mag_plate_depth, 1, 1,
                     beads_wash_tfer_vol_ul, 1,source_pep_flow_plate, pep_pos,
source_pep_flow_plate_depth, 0)

            if use_new_tip == 1:
                pip300.drop_tip()
            else:
                pip300.return_tip()

        # endregion

        # region add elution buffer, brief mixing

        tip_col = change_tipRack_func(tip_col, 1)
        tip_pos = "A" + str(tip_col)
        pip300.pick_up_tip(tip_rack_300[tip_pos])

        if prev_elution_channel_incr > change_elute_channel_every_n_iteration:
            prev_elution_channel_incr = 1
```

```
            elute_channel_incr = elute_channel_incr + 1
            elute_pos = "A" + str(elute_channel_incr)

        pip300.transfer(elute_tfer_vol_ul,
                        source_buf_plate[elute_pos].top(z=source_buf_plate_depth),
                        dest_mag_plate[mag_col].top(z=-1),
                        new_tip='never', blow_out=False)

        mix_func(mix_mag_brief_elu_cycle, elute_tfer_vol_ul, dest_mag_plate,
mag_col, dest_mag_plate_depth_mix,
                 dest_mag_plate_depth, 1, 0, 0, 0, "0", 0, 0, 0)

        if use_new_tip == 1:
            pip300.drop_tip()
        else:
            pip300.return_tip()

        prev_elution_channel_incr = prev_elution_channel_incr + 1

        # endregion

    # endregion

    # region elution


    for e in range(elute_cycle):

        for d in dest_mag_cols_array:
            mag_col = "A" + d
            pep_pos = "A" + d
```

```
            tip_col = change_tipRack_func(tip_col, 1)
            tip_pos = "A" + str(tip_col)
            pip300.pick_up_tip(tip_rack_300[tip_pos])

            elute_tfer_vol_plus_air_gap_ul = elute_tfer_vol_ul + 5.5 + air_gap_ul

            # mixing for elution
            mix_func(mix_mag_elu_cycle, elute_tfer_vol_ul, dest_mag_plate, mag_col,
dest_mag_plate_depth_mix,
                     dest_mag_plate_depth, 1, 0, 0, 0, "0", 0, 0, 1)

            mag_mod.engage(height_from_base=magnet_height)
            ctx.delay(seconds=pause_pip_sec)

            # region wash tip

            pip300.mix(mix_mag_activate_cycle_2, elute_tfer_vol_ul,
                       dest_mag_plate[mag_col].top(z=dest_mag_plate_depth_mix))
            pip300.blow_out(dest_mag_plate[mag_col].top(z=0.1))
            ctx.delay(seconds=pause_pip_sec_tipWash)

            # endregion

            # region transfer elution to a new 96-well plate

            final_tfer_elu_vol = elute_tfer_vol_ul + 5.5

            pip300.aspirate(final_tfer_elu_vol,
dest_mag_plate[mag_col].top(z=dest_mag_plate_depth))
            ctx.delay(seconds=pause_pip_sec_asp)
```

```
    #pip300.air_gap(air_gap_ul)

    pip300.dispense(elute_tfer_vol_plus_air_gap_ul,
                    dest_elute_plate[pep_pos].top(z=dest_elute_plate_depth))

    # endregion

    # region add fresh elution buffer, brief mixing

    if e == (elute_cycle - 1):
        # mix in the elution plate
        pip300.mix(elute_final_mix_cycle, mix_set_vol,
                   dest_elute_plate[pep_pos].top(z=dest_elute_plate_depth))

        pip300.blow_out(dest_elute_plate[pep_pos].top(z=0.01))

        if use_new_tip == 1:
            pip300.drop_tip()
        else:
            pip300.return_tip()

        mag_mod.disengage()

    else:
        pip300.blow_out(dest_elute_plate[pep_pos].top(z=0.01))

        if use_new_tip == 1:
            pip300.drop_tip()
        else:
            pip300.return_tip()
```

```
            mag_mod.disengage()

            tip_col = change_tipRack_func(tip_col, 1)
            tip_pos = "A" + str(tip_col)
            pip300.pick_up_tip(tip_rack_300[tip_pos])

            if prev_elution_channel_incr > change_elute_channel_every_n_iteration:
                prev_elution_channel_incr = 1
                elute_channel_incr = elute_channel_incr + 1
                elute_pos = "A" + str(elute_channel_incr)

            pip300.transfer(elute_tfer_vol_ul,
                            source_buf_plate[elute_pos].top(z=source_buf_plate_depth),
                            dest_mag_plate[mag_col].top(z=-1),
                            new_tip='never', blow_out=False)

            mix_func(mix_mag_brief_elu_cycle, elute_tfer_vol_ul, dest_mag_plate,
mag_col,
                    dest_mag_plate_depth_mix,dest_mag_plate_depth, 1, 0, 0, 0, "0", 0,
0, 0)

            if use_new_tip == 1:
                pip300.drop_tip()
            else:
                pip300.return_tip()

            prev_elution_channel_incr = prev_elution_channel_incr + 1
        # endregion
```

```
        if e != (elute_cycle - 1):
            ctx.delay(seconds=pause_btw_elu_sec)
        else:
            ctx.pause('''Add elution buffer for beads collection, click Resume''')

            tip_col = change_tipRack_func(tip_col, 1)
            tip_pos = "A" + str(tip_col)
            pip300.pick_up_tip(tip_rack_300[tip_pos])

            collectBead_pos = "A" + str(source_collect_bead_start_col)

            for d in dest_mag_cols_array:
                mag_col = "A" + d

                pip300.transfer(collect_bead_tfer_vol_ul,
source_buf_plate[collectBead_pos].top(z=source_buf_plate_depth),
                                dest_mag_plate[mag_col].top(z=-1),
                                new_tip='never', blow_out=False)

                pip300.blow_out(dest_mag_plate[mag_col].top(z=0.1))

            if use_new_tip == 1:
                pip300.drop_tip()
            else:
                pip300.return_tip()



    return tip_col

    # endregion
```

```
# endregion



tip_incr = rack300_start_col

# region Step 1: remove wash buffer from all

tip_incr = removeWashBuffer_beforeBind_func(tip_incr)

# endregion

ctx.pause('''Transfer sample, click Resume''')

# region Step 2: add samples

tip_incr = addPep_func(tip_incr)

# endregion

ctx.pause('''Incubation, click Resume''')

# region Step 3: incubation by pipette mixing

for incub in range(incubation_mix_mag_cycle):
    tip_incr =  Pep_incubation_func(tip_incr)

    if incub < (incubation_mix_mag_cycle-1):
        ctx.delay(seconds=pause_btw_sec)
```

```
# endregion

ctx.pause('''Collect flow through, wash, and elution, click Resume''')

# region Step 4: collect flowthrough, wash, and elution

tip_incr = wash_elution_afterBind_func(tip_incr, 1, 0)

# endregion

ctx.comment('''Process Complete.''')
```
